# Ewing Sarcoma Ewsa Protein Regulates Chondrogenesis of Meckel’s Cartilage through Modulation of Sox9 in Zebrafish

**DOI:** 10.1371/journal.pone.0116627

**Published:** 2015-01-24

**Authors:** Chris Merkes, Timothy K. Turkalo, Nicole Wilder, Hyewon Park, Luke W. Wenger, Seth J. Lewin, Mizuki Azuma

**Affiliations:** Molecular Biosciences, University of Kansas, 7031 Haworth, 1200 Sunnyside Avenue, Lawrence, KS 66045, United States of America; Rutgers-Robert wood Johnson Medical School, UNITED STATES

## Abstract

Ewing sarcoma is the second most common skeletal (bone and cartilage) cancer in adolescents, and it is characterized by the expression of the aberrant chimeric fusion gene *EWS/FLI1.* Wild-type EWS has been proposed to play a role in mitosis, splicing and transcription. We have previously shown that EWS/FLI1 interacts with EWS, and it inhibits EWS activity in a dominant manner. Ewing sarcoma is a cancer that specifically develops in skeletal tissues, and although the above data suggests the significance of EWS, its role in chondrogenesis/skeletogenesis is not understood. To elucidate the function of EWS in skeletal development, we generated and analyzed a maternal zygotic (MZ) *ewsa/ewsa* line because the *ewsa/wt* and *ewsa/ewsa* zebrafish appeared to be normal and fertile. Compared with *wt/wt,* the Meckel’s cartilage of MZ *ewsa/ewsa* mutants had a higher number of craniofacial prehypertrophic chondrocytes that failed to mature into hypertrophic chondrocytes at 4 days post-fertilization (dpf). Ewsa interacted with Sox9, which is the master transcription factor for chondrogenesis. Sox9 target genes were either upregulated (*ctgfa, ctgfb, col2a1a,* and *col2a1b*) or downregulated (*sox5, nog1, nog2,* and *bmp4*) in MZ *ewsa/ewsa* embryos compared with the wt/wt zebrafish embryos. Among these Sox9 target genes, the chromatin immunoprecipitation (ChIP) experiment demonstrated that Ewsa directly binds to *ctgfa* and *ctgfb* loci. Consistently, immunohistochemistry showed that the Ctgf protein is upregulated in the Meckel’s cartilage of MZ *ewsa/ewsa* mutants. Together, we propose that Ewsa promotes the differentiation from prehypertrophic chondrocytes to hypertrophic chondrocytes of Meckel’s cartilage through inhibiting Sox9 binding site of the *ctgf* gene promoter. Because Ewing sarcoma specifically develops in skeletal tissue that is originating from chondrocytes, this new role of EWS may provide a potential molecular basis of its pathogenesis.

## Introduction

*EWS* (EWSR1, Ewing sarcoma breakpoint region 1) was originally discovered in Ewing sarcoma, the second most common bone cancer in adolescents and young adults. Ewing sarcoma cells display undifferentiated morphology known as small round blue cell, suggesting that the impairment of skeletal lineage differentiation may contribute to its pathogenesis. Currently, there is little knowledge of any correlation between the differentiation of skeletal elements and Ewing sarcoma formation. A major genetic hallmark of Ewing sarcoma is the aberrant fusion gene *EWS/FLI1*. This fusion gene is generated by chromosomal translocation, and it contains a sequence coding the N-terminal transactivation domain of EWS fused to the C-terminal domain of the ETS transcription factor FLI1 [1]. EWS/FLI1 has been shown to function as a transcription factor, and it leads to the transcriptional misregulation of its target genes [2–8]. EWS/FLI1-dependent transcriptional activity is enhanced by interactions with RNA helicase A [9,10]. A subgroup of miRNAs was reported to regulate EWS/FLI1, and this regulates the insulin-like growth factor (IGF) pathway [11]. During development, FLI1 transcriptionally regulates hemangioblast differentiation [12]. EWS plays roles in transcription, splicing and mitosis [13–17].

Compromising the function of EWS may play a significant role in Ewing sarcoma formation. First, it was reported that EWS/FLI1 interacts with wild-type EWS [18,19]. As a result, this interaction leads to the inhibition of EWS activity in a dominant manner, and it induces aberrant transcription, splicing, or induces mitotic dysfunction [13,17,19]. Second, only a single EWS allele is retained in Ewing sarcoma cells due to the generation of EWS/FLI1 by chromosomal translocation, which may induce haploinsufficiency. Understanding how these reported functions of EWS are regulated in bones and cartilage cells is particularly important because it will supply a platform to study molecular pathways in Ewing sarcoma cells.

A study utilizing EWS knockout mice revealed that a homozygous EWS-/- genotype is embryonic lethal in mice with 129SvEv and C57BL/6 backgrounds and that 90% of EWS knockout mice with the black Swiss background die after birth. The same study indicated that the surviving offspring from the black Swiss background display senescence, failure in B cell differentiation and sperm meiosis, thereby demonstrating the role of EWS in recombination [20]. Although the cause of death was undetermined, the EWS knockout mice were smaller in size compared with their wildtype littermates, indicating that EWS may play a role in development. Previously, we identified two homologs of the human *EWS* gene in zebrafish: *ewsa* and *ewsb* [16]. The gene duplication of zebrafish often has a redundant role, thus providing an attractive resource to elucidate the early developmental stage because mutants often display a milder phenotype. In addition, the molecular function is well conserved among vertebrates. For these reasons, we utilized an *ewsa* null mutant zebrafish enabling the observation of their development from the one-cell stage because they spawn eggs *ex vivo*. In this study, we discovered that the maternal zygotic (MZ) *ewsa/ewsa* mutant zebrafish display defects in chondrogenesis, and sought to address the molecular function of Ewsa.

The craniofacial skeleton/cartilage is primarily derived from neural crest cells. Neural crest cells are a unique multipotent cell population that gives rise to multiple lineages, including craniofacial bones, pigment cells, and peripheral nerves. After neural tube closure, cranial neural crest cells undergo the epithelial-mesenchymal transition (EMT), and the mesenchymal cells migrate ventrally to populate a subset of pharyngeal arches [21–23]. These arch cells receive patterning signals from *dlx* gene expression and migrate further to form mesenchymal condensations that give rise to the craniofacial cartilages that ultimately form the craniofacial bones [24]. Endochondral ossification is one of the major mechanisms of skeletogenesis [25]. Endochondral ossification is a multi-step process that results in the formation of long bones and involves the following steps: 1) migration and condensation of mesenchymal cells; 2) differentiation from mesenchymal cells to prehypertrophic chondrocytes; 3) secretion of extracellular matrix components; 4) differentiation from prehypertrophic chondrocytes to hypertrophic chondrocytes; and 5) formation of mature calcified bones.

Importantly, differentiation of the craniofacial skeleton and vertebrae is regulated by the master transcription factor, Sex-determining Region Y (SRY) box 9 (SOX9) [26,27]. A heterozygous mutation of SOX9 leads to campomelic dysplasia (CD), a syndrome that is characterized by defective chondrogenesis and sex reversal. SOX9 is a master regulator of chondrogenesis because SOX9 -/- mice fail to form cartilage [28]. The target genes of SOX9 (e.g., *pax1/pax9*) are known to regulate skeletogenesis, and the knockdown or knockout mutants of these genes often result in a reduction of appendages, body regions, or the entire animal [26,27,29,30]. To undergo endochondral ossification, craniofacial prehypertrophic chondrocytes and vertebral condensations require transcriptional activation of Sox9 to regulate their proliferation, maturation of hypertrophic chondrocytes, and expression of extracellular matrix proteins. Numbers of Sox9 target genes have been reported including *SOX5*, *AGGRECAN*, *COLLAGEN TYPE IIa1 and CONNECTIVE TISSUE GROWTH FACTOR (CTGF)* [26,28,31]. High level of expression of Sox9 protein in proliferating and prehypertrophic chondrocytes is downregulated in hypertrophic chondrocytes [26,32–34]. CTGF also plays a significant role in the transition from prehypertrophic chondrocytes to hypertrophic chondrocytes. The knockout mice of *CTGF* displayed an expansion of hypertrophic chondrocytes, whereas transgenic mice of *CTGF* displayed a reduction of hypertrophic chondrocytes [35]. In this study, we demonstrated that impaired differentiation of prehypertrophic chondrocytes into hypertrophic chondrocytes in Meckel’s cartilage of MZ *ewsa/ewsa*. This phenotype was possibly induced by aberrant upregulation of *ctgfa* and *ctgfb* through Ewsa modulation of Sox9-binding site of its promoter. This study is the first demonstration of a role for Ewing sarcoma Ewsa protein in chondrogenesis/skeletogenesis. The EWS-dependent regulation of SOX9 may also provide a platform for dissecting not only the role of EWS/FLI1 but also the development of Ewing sarcoma.

## Experimental Procedures

### Ethics statement

All of the zebrafish were maintained and humanely euthanized following the protocols approved by the University of Kansas, Lawrence Institutional Animal Care and Use Committee (IACUC) (Animal use statement: Permit Number #197–01).

### Zebrafish maintenance

The *ewsa* mutant line was generated via virus-mediated insertional mutagenesis by Znomics, Inc. Oregon AB* and *ewsa* mutant zebrafish lines were maintained at 28°C using an automatic filtration system from Aquatic Habitats. All of the embryos were staged as previously described [36].

### Alizarin red staining

Adult zebrafish were stained with alizarin red as previously described [37]. The images were documented with a Leica DFC320 camera mounted on a Leica MZ FLIII dissecting microscope.

### Alcian blue staining

The embryos were stained with alcian blue as previously described with minor modifications. The embryos were equilibrated in acidified ethanol (5% HCl, 70% ethanol) before staining overnight in 0.1% alcian blue dissolved in acidified ethanol [38]. The specimens were then washed with acidified ethanol and dehydrated with an ethanol series before transfer to glycerol. Microdissections and flat mounting were performed using tungsten needles as previously described [37]. The images were documented with a Leica DFC320 camera mounted on a Leica MZ FLIII dissecting microscope.

### Immunohistochemistry

Immunohistochemistry of the zebrafish embryos was performed as previously described with minor modifications [16]. N-terminus of *ewsa* (1–762bp) was cloned into pET28a vector, and the Ewsa recombinant protein was purified from *Escherichia coli (E*. *coli)*. Using the Ewsa protein, polyclonal antibody was generated in rabbit (Pacific Immunology, CA). The following antibodies were used in this study: anti-Collagen II primary antibody (Developmental Studies Hybridoma Bank at University of Iowa, IA) (1:250), anti-Ihh (1:100)(Abcam, MA), anti-Collagen X (1:100)(Abcam, MA), anti-Sox9 (1:100)(Abcam, MA), anti-Ewsa (1:1000)(generated using the recombinant Ewsa proteins against 1—254 amino acid in rabbit in our laboratory), anti-mouse Alexa 594 secondary antibody (1:250) and anti-rabbit Alexa 488 secondary antibody (1:250). The images were documented with an Exi Aqua camera (Q Imaging) mounted on an Eclipse Ti microscope (Nikon).

### *In situ* hybridization

*In situ* hybridization was performed as previously described [39,40].

### Co-immunoprecipitation (co-IP)

The co-IP experiment for zebrafish Ewsa and Sox9 was performed with 27 hpf zebrafish embryos. One hundred fifty zebrafish embryos were humanely euthanized and lysed with 500 μl of lysis buffer (2 mM Tris pH 7.6, 1% Triton X-100, 1 mM EDTA, 150 mM NaCl) by incubation on ice for 20 minutes. The lysates were collected after centrifugation (13,400 rcf for 15 minutes). Lysis buffer (1.5 mL) was added to the supernatant, and the lysates were incubated with an anti-mouse IgG nonspecific control and anti-SOX9 antibodies (AbCam, MA) conjugated to DYNA-protein G beads (Invitrogen, NY) at 4°C for 1 hour. The co-IP experiment for human EWS and SOX9 was performed with HeLa cells. HeLa cells with 90% confluency in 9-cm dishes were transfected with 26.4 µg of PSG5 FLAG tagged-SOX9 or PSG5 empty vector using Lipofectamine 2000 (Invitrogen, NY). Sixteen hours after transfection, the cells were washed twice with phosphate buffered saline (PBS) and lysed with 3 mL of lysis buffer (2 mM Tris pH 7.6, 1% Triton X-100, 1 mM EDTA, 150 mM NaCl) by incubation on ice for 20 minutes. The lysates were collected after centrifugation (13,400 rcf for 1 minute). An aliquot (900 μL) of the lysates was incubated with an anti-mouse IgG nonspecific control and anti-FLAG antibodies (Agilent Technologies, CA) conjugated to DYNA-protein G beads (Invitrogen, NY) at 4°C for 1 hour. The immunoprecipitated samples obtained from the zebrafish embryos and HeLa cells were washed with lysis buffer (rotating at 4°C) for 20 minutes 3 times and soluble denatured protein was obtained by adding 1x SDS sample buffer at 92°C for 5 minutes. The immunoprecipitated samples were subjected to western blot analysis using anti-Ewsa (made by our laboratory) and anti-SOX9 (AbCam, MA) antibodies for the zebrafish samples and anti-EWS (SantaCruz, CA) or anti-FLAG (Agilent Technologies, CA) antibodies for the HeLa cell samples. All of the western blots were visualized using the SuperSignal West Femto Chemiluminescent Substrate (Pierce, IL).

### qPCR

Total RNA was isolated from 27 hpf embryos using RNeasy Mini Kit (Qiagen, MD) following the manufacturer’s protocol and using the optional on-column DNase digestion (Qiagen, MD). One microgram of RNA was subjected to 20 μL of a cDNA synthesis reaction using oligo dT primers and SuperScript III Reverse Transcriptase (Invitrogen, NY). Then, qPCR was performed using 1 μL of cDNA or control samples in 20 μL of total reaction solution with 1x Power SYBR Green PCR Master-Mix (Applied Biosystems, Invitrogen, NY) and 0.9 μM forward and reverse primers.

*Bmp4* (CCTGGTAATCGAATGCTGATG & CGCTTTCTTCTTCCCTTCCTC),

*Cata1* (GGGATCCCAAGAGTTTGGAG & GATGGACCTTTGTTGCTGGAG),

*Cata2* (GACGAGCGCTACGTCCCC & CTGTGTGACCAGCGGCTCC),

*Ctsb* (CGGCTGGCGTTCCTGTGTG & GACCATCTCATGGGACAAGG),

*Col1a1a* (GCTTTGTGGATATTCGGCTGG & CCAATGTGCAGCTGCCGCC),

*Col2a1a* (GCTGGATTCACGGACTCTCC & CCTTTGCACCAAGTGACCGG),

*Col2a1b* (GGAGCAAGACCCCGGCGG & GCCGCTGTCACACACACAG),

*Col9a* (GCTGAGTTCTTCATCGTCCTC & CAGCGGGGCCTTGAGCTC),

*Col11a1a* (GAGAGGCCAAGGTGGTCCC & CCTGCAGAACCAGGACGAG),

*Col11a1b* (GTGGTCCACGAGATGGAAAAC & GCTCTCACTTGTGTTGCCTG),

*Col11a2* (GATATTCGGAAGAAGCGGAGG & CGCAAAACATCTACTGGATCTG),

*Ctgfa* (GTGTGATTGCTCTGCTGTTCC & GGTGAACACTGGGGCGGC),

*Ctgfb* (CTGGAACAGCATTCACCAGAG & CTCGTCTGGGCAATCACAGG),

Epyc (CTCCCCCGAGATACGTCCG & CCTCGTAGGCATTGTCGCC),

*Erk1* (GCGGAATCGGGCAGTAGCG & CGCTTGGTCCCCCAGGTG),

*Fmoda* (GCGGCTAATTGCTCTCCTGC & CATAACCCCGGCTGTGTAAAG),

*Gapdh* (CGGATTCGGTCGCATTGGC & GGTCATTGATGGCCACGATC),

*Grb10a* (CCTTAGCTGGATGTCCAGAC & GGATGGTCTGTGAGATGAGG),

*Igf2r* (GTCGTGTTGGATTTTGCGGAG & GGCTGTCATCCGAAGCCGC),

*IL1* (GCATGCGGGCAATATGAAGTC & GCGGATCTGAACAGTCCATC),

*Lef1* (CGCAGTTGTCAGGTGGAGG & GCTCCTTGTGCGGGTCTCC),

*Matn4* (GTGTTGGTGTATGTCAGTGTG & GTCAACCGGGCCAGATTTAC),

*Nog1* (GTTTGCTGTCCGCGTACTTG & GCTCCAGCAGGGGTAAAGTG),

*Nog2* (CTACTGCTGCTCCTGTGCG & CTCGATGAGGTCTGGGACG),

*Ptch1* (CTCGGCTGTTAATGTCTCCTC & CGATAGTTGCCCCTATTTCTC),

*Ptch2* (GCCGCCTGTGAACTCAGATC & CTTTCTGTCCCACAGCTTTCC),

Prkacaa (GCCAAGAACAAGGGCAATGAG & CAGTGTTCTGTGCTGGGTTC),

Prkacab (GGGGCAACGAAATGGAAAGC & CCAGGCAGGCGGTGTTCTG),

Prelp (GCTGGGCTTGCATACTGCTG & GATGGGCGAACAGGCTTTGG),

*Runx2b* (CATTCCCGTAGATCCCAGCG & CTGCTGAGGTCCTGCATTCG),

*Sox5* (CTTACTGAGCCTGAGCTTCC & CGTCGCCATGACTACCTCTC),

*Sox9a* (GACCCCTACCTGAAGATGAC & CGCGGAGTCCTCGGACATG),

*Sox9b* (GAAGATGAGTGTGTCCGGAG & GTCTCGCTGTCCGATCCCG),

*Stat1a* (CTCAGTGGTTGGAGCTTCAG & CTGAGATATTGTCGGATGGCC),

*Stat1b* (GCTCTGGAACCAGCTGCAG & GTCGGATCTCCATTGGGAAAG),

*Sdc3* (GCTCCCGTGCTGGATAACG & CTCATCTCCAGAGCTCTCATC),

*Tgfb3* (GCAAAGGACTGCTGTTTGTTC & CAATGTCCACTGTGGTGCAG),

*Vegfaa* (GCGTGCAAGACCCGAGAGC & GCGCATGAGAACCACACAGG),

*Vegfab* (CTTTGCTGTTCGCGTGCTCC & GTGCTTCTGCCTCCCTCTC).

Ninety-six-well plates were run on a StepOnePlus Real-Time PCR System (Applied Biosystems, Invitrogen, NY), and the cycle thresholds were determined using the manufacturer’s software. The relative expression levels were calculated using the comparative ddCT method and the GAPDH mRNA level as the endogenous control [41].

### Chromatin Immunoprecipitation (ChIP)

Thirty-five of 27hpf *wt/wt* embryos were euthanized and subjected to ChIP assay as described before [42]. The immunoprecipitated DNA using anti-Ewsa antibody were subjected to PCR using the following forward and reverse primers.

*ctgfa* (TAGAACCATACCACACCTGC & AACTTGCAGGTCATGTTTTACAC),

*ctgfb* (GTCTAGCATGACATCATGCG & TGTAGGCCAGTCTGCTGGG),

### Statistical Analysis

Statistical analysis was performed using R (version 3.0.0). Fisher’s exact test was used to compare the incidences of normal or abnormal bone and cell morphologies between the wild type and mutant zebrafish. Welch’s t-test was used to compare GAPDH-normalized relative expression of mRNAs obtained by qPCR between the wild type and mutant zebrafish.

## Results

### Ewsa promotes the differentiation of prehypertrophic chondrocytes into hypertrophic chondrocytes in Meckel’s cartilage

**Figure 1:**
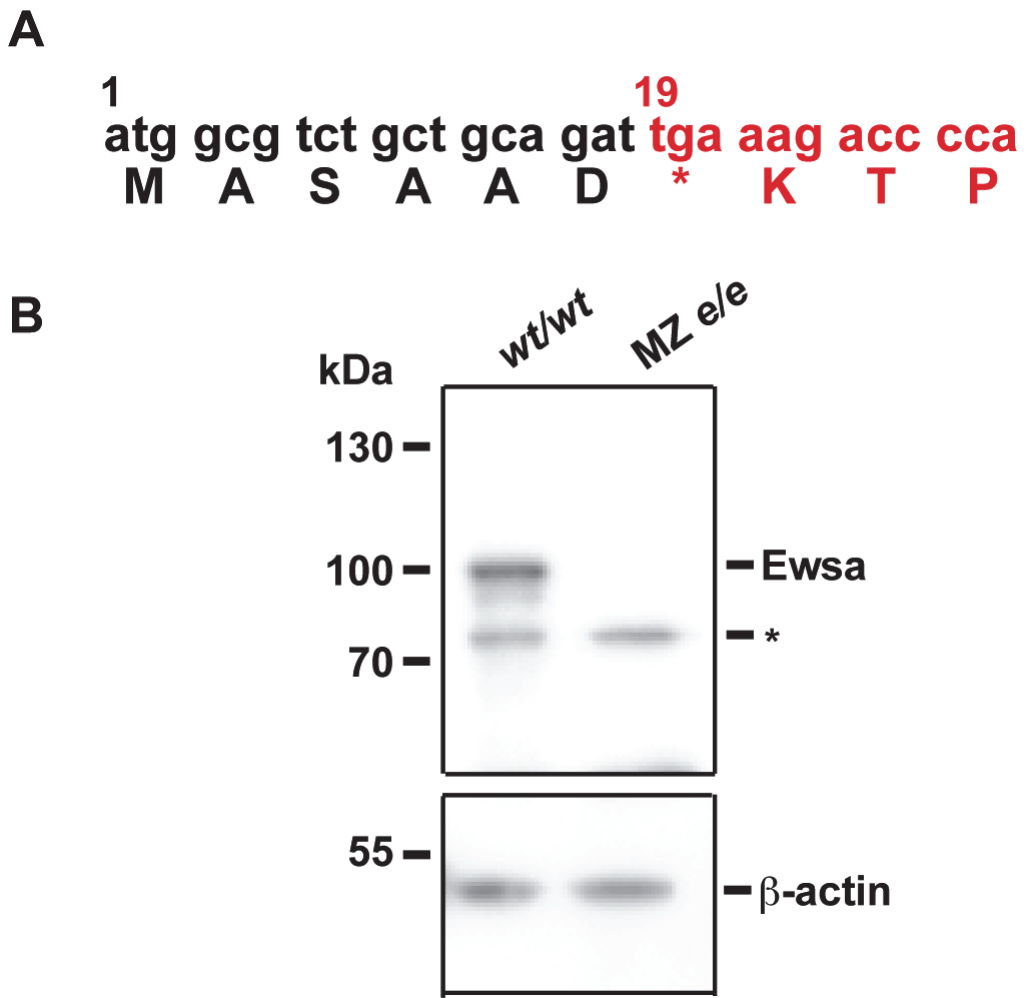
MZ *ewsa/ewsa* mutant is a null mutant. **A.** Schematic of the insertion site of the *ewsa* gene. After the 6^th^ amino acid, a stop codon was introduced. Zebrafish *ewsa* gene (black), virus sequence (Red). **B.** Western blot of zebrafish cell lysates obtained from the *wt/wt* and MZ *ewsa/ewsa* embryos at 6dpf. Top panel: probed with the anti-Ewsa antibody (Ewsa, Ewsa protein; *: non-specific protein), bottom panel: probed with β-actin antibody.

Previous reports have shown that EWS-/- homozygosity in 129SvEv or C57BL/6 inbred mouse backgrounds is lethal to embryos. However, 90% of EWS-/- knockout mice in the black Swiss outbred line survive to birth but die before weaning, with only 1 of 18 surviving to postnatal day 21 [20]. These results indicate that Ews plays a significant role in early development. To address the role of Ews during early development, we utilized a zebrafish model. Due to the genome duplication event before teleost radiation, there are two zebrafish genes, termed *ewsa* and *ewsb*, which are homologous to human *EWS* [16]. Zebrafish provides an attractive model because mutants often display a milder phenotype due to a redundant role between two duplicated genes. For this reason, we obtained and analysed an *ewsa* mutant zebrafish line originally isolated in an insertional mutagenesis screen (Znomics Inc.). All of the heterozygous *ewsa/wt* and homozygous *ewsa/ewsa* zebrafish appeared to be normal and fertile. We further generated a MZ *ewsa/ewsa* mutant line by intercrossing zygotic *ewsa/ewsa* homozygous mutants. A retroviral vector was integrated immediately after the eighteenth base pair (sixth amino acid) from the start codon, and its insertion generated a stop codon (Fig. 1A). To confirm the knockout of the Ewsa protein, we generated an anti-Ewsa antibody in rabbits against a protein containing amino acids 1—254 that was purified from *Escherichia coli* (*E*. *coli)*. Using this antibody, we performed a western blot using the lysates extracted from 6 dpf *wt/wt* and MZ *ewsa/ewsa* embryos. The Ewsa protein was absent from the protein extracts from the *ewsa/ewsa* mutants, thereby confirming a null Ewsa protein mutant (Fig. 1B).

Unlike EWS-/- mice, which have a high postnatal lethality, the MZ *ewsa/ewsa* zebrafish mutants survive until adulthood and are fertile. The phenotypic discrepancy between the EWS-/- mice and *ewsa/ewsa* mutant zebrafish may be due to the redundant expression of the *ewsb* gene in zebrafish. The MZ *ewsa/ewsa* mutants developed with normal morphology, including a straight notochord and somites at the 17 somite stage (S1A Fig.). To examine whether specification was affected during development, *in situ* hybridizations were performed in *wt/wt* and MZ *ewsa/ewsa* mutant embryos at 27 hours post-fertilization (hpf). Three probes for tissue-specific genes, including *shh* (notochord), *eng3* (midbrain-hindbrain boundary), *krox20* (rhombomere 2 and 4 in hindbrain) and *wnt1*, did not exhibit any significant differences in their expression pattern between the *wt/wt* and MZ *ewsa/ewsa* mutants, thereby indicating that the overall dorsal-ventral and anterior-posterior patterning, including the brain and notochord, were not affected in the mutant (S1B Fig.). We previously observed a high level of apoptotic cell death in the *ewsa* and *ewsb* morpholino-injected embryos. However, we did not observe a high level of cell death in the brain region of *ewsa/ewsa* mutant embryos. The discrepancy of the phenotype may be a result of morpholino-induced cell death due to off-target effects [43].

**Figure 2:**
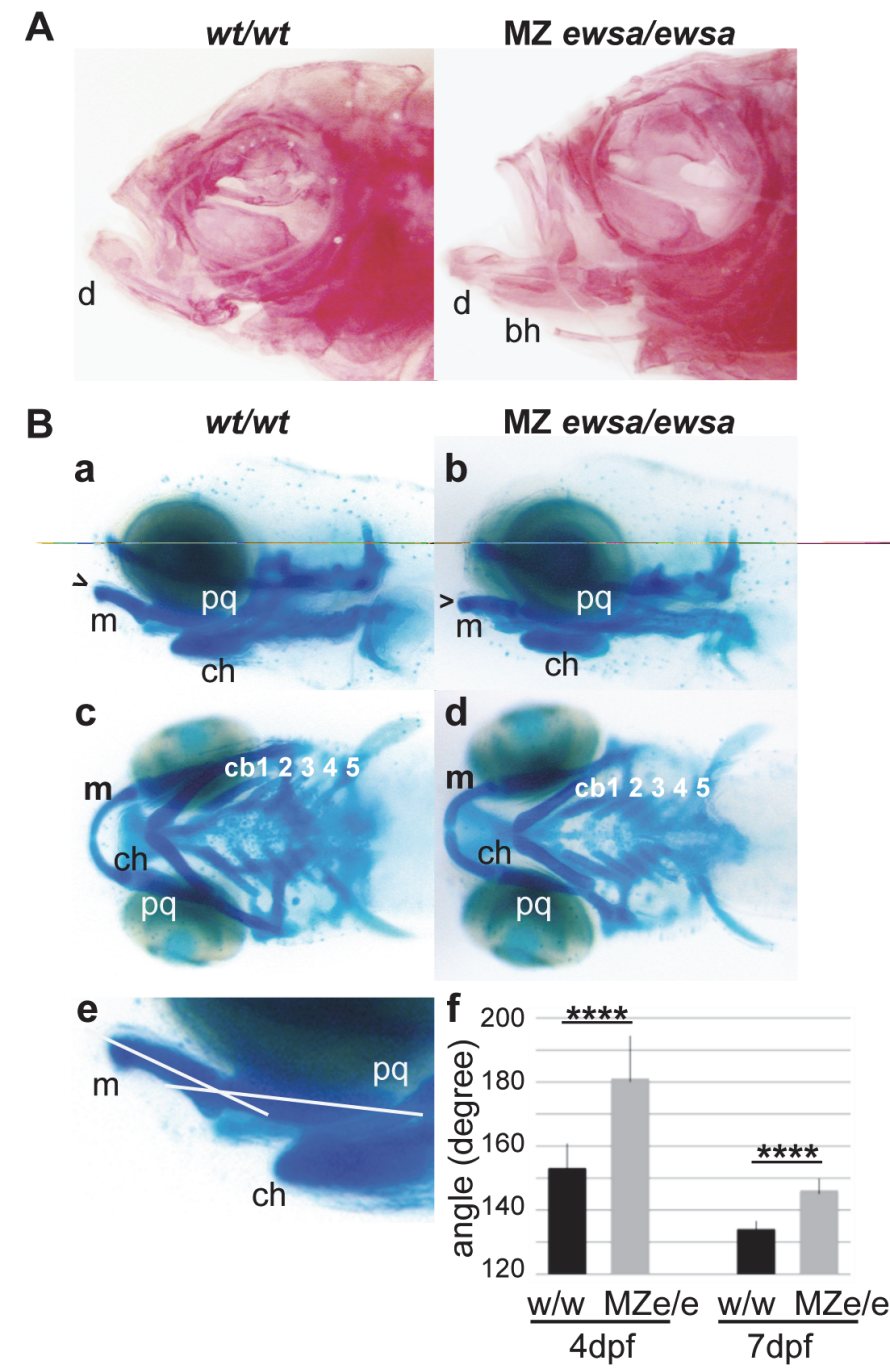
4 dpf MZ ewsa/ewsa mutants display an aberrant angle of Meckel’s cartilage and palatoquadrate. **A.** Lateral views (anterior to the left) of *wt/wt* (left) and MZ *ewsa/ewsa* (right) and ventral views of adult zebrafish. The calcified bones were visualized by alizarin red staining. **B.** Lateral views (anterior to the left) of (a) *wt/wt* and (b) MZ *ewsa/ewsa* and ventral views of (c) *wt/wt* and (d) MZ *ewsa/ewsa* chondrocytes from 4 dpf zebrafish embryos visualized with alcian blue. (e and f) Angle formed by Meckel’s cartilage showing that the palatoquadrate is wider in the MZ *ewsa/ewsa* mutant than *wt/wt* at 4 dpf and 7 dpf. bh: basihyal, d: dentary, m: Meckel’s cartilage, pq: palatoquadrate, ch: ceratohyal, cb: ceratobranchial.

The MZ *ewsa/ewsa* mutant zebrafish were raised until adulthood to determine whether development was affected. The adult MZ *ewsa/ewsa* mutant zebrafish displayed protruding jaws and curved axial skeleton, suggesting that Ewsa plays a role in skeletogenesis (data not shown). To further investigate the morphological changes in MZ *ewsa/ewsa* mutant zebrafish, the skeletal elements were visualized with alizarin red staining (Fig. 2A). The dentary (the dermal bone that forms the lower jaw) protruded anteriorly in the adult MZ *ewsa/ewsa* mutants (65%; n = 20 fish) compared to the adult *wt/wt* zebrafish (0%; n = 11 fish) (Fisher’s exact test, p = 0.0004) (Fig. 2A). In addition, the basihyal bones of the MZ *ewsa/*ewsa mutants projected ventrally resulting in misalignment with the lower jaw line (40%; n = 20) (Fig. 2A). In contrast, none of the *wt/wt* zebrafish displayed misalignment of the basihyal bones (0%; n = 11) (Fisher’s exact test, p = 0.03) (Fig. 2A). To determine when the MZ *ewsa/ewsa* mutants started to display skeletal defects, we investigated the formation of chondrocytes because these bones are derived from chondrocytes. The chondrocytes in the *wt/wt* and MZ *ewsa/ewsa* mutants at 2 to 9 dpf were visualized using alcian blue. The MZ *ewsa/ewsa* mutant zebrafish displayed no apparent phenotypic differences in craniofacial chondrocytes between the *wt/wt* and MZ *ewsa/ewsa* mutants till 3 dpf. At 4 dpf, the MZ *ewsa/ewsa* mutants displayed an aberrant angle of Meckel’s cartilages compared to the *wt/wt* embryos (Fig. 2B a and b). The ventral images of the embryos revealed that the overall patterning of the craniofacial chondrocytes was not affected (Fig. 2B c and d). Lateral view images of the embryos were acquired, and the angles formed by Meckel’s cartilage and palatoquadrate (pq) were measured from the photographs using the Image J software (Fig. 2B e). The average angles in the MZ *ewsa/*ewsa mutants (4 dpf, 181± 14^o^ and n = 32; and 7 dpf, 146± 4^o^ and n = 14) were wider than those in the *wt/wt* fish (4 dpf, 153± 8^o^ and n = 28; and 7 dpf, 134± 3^o^ and n = 7) (Welch’s t-test; 4 dpf, p<10^–12^; and 7 dpf, p<10^–4^) (Fig. 2B f).

**Figure 3:**
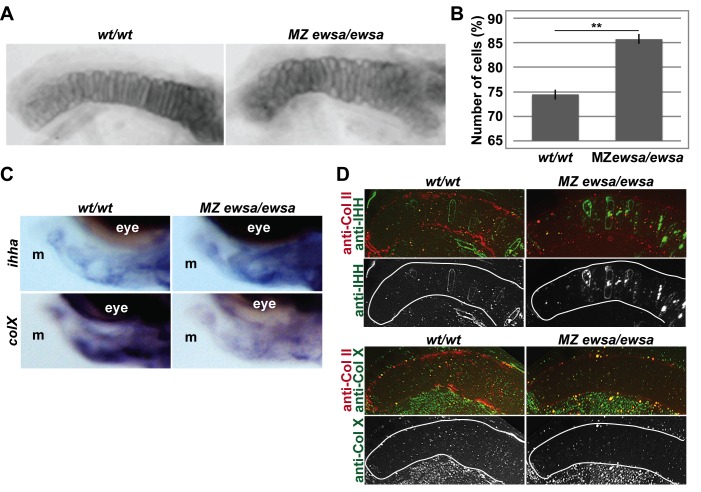
Prehypertrophic chondrocytes of Meckel’s cartilage in MZ *ewsa/ewsa* fails to differentiate into hypertrophic chondrocytes. **A.** Flat mounted Meckel’s cartilage of 4 dpf (left) *wt/wt* and (right) MZ *ewsa/ewsa*. **B.** Number of cells of Meckel’s cartilage of 4 dpf *wt/wt* and MZ *ewsa/ewsa* zebrafish (P = 0.0002). **C.** Lateral views (anterior to the left, dorsal to the top) of 4dpf (Left) *wt/wt* and (Right) MZ *ewsa/ewsa* visualized by *in situ* hybridization using probe for *ihha* (top panel) and *colX* (bottom panel). m: Meckel's cartilage. **D.** Ventral views (anterior to the left) of Meckel's cartilage of (Left) *wt/wt* and (Right) MZ *ewsa/ewsa* visualized by immunohistochemistry using (top) anti-IHH antibody (green), and anti-Collagen type II antibody (Red) (4dpf), and (bottom) anti-Collagen X antibody (green) and anti-Collagen type II antibody (Red) (5dpf).

The 4 dpf embryos were micro-dissected, and the craniofacial cartilages were flat mounted. The overall chondrocyte patterning of the bones was not affected, except the shape of Meckel’s cartilage in the 4 dpf MZ *ewsa/*ewsa mutants (S2 Fig. and Fig. 3A). Because the defects of Meckel’s cartilage in the MZ *ewsa/ewsa* mutant was the earliest major phenotype we observed, we further focused to elucidate the molecular function of Ewsa during formation of Meckel’s cartilage. However, it is noteworthy that there is a possibility of other cartilage structure of the mutant may be affected in later stages. Previous study showed that the chondrocytes of Meckel’s cartilage at 5dpf displays hypertrophic cell morphology [44]. For this reason, we examined earlier stages of Meckel’s cartilage cell morphology, and 4dpf was the critical stage for transition from small and round prehypertrophic chondrocytes to long and columnar hypertrophic chondrocytes with aligned columnar cells. All of the dissected MZ *ewsa/ewsa* embryos (n = 14) displayed disorganized, round and small cells in Meckel’s cartilage, but none of the *wt/wt* embryos displayed these defects (n = 14) (Fisher’s exact test, p = 0.0002)(Fig. 3A). We further counted the number of chondrocytes in Meckel’s cartilage, and the 4 dpf MZ *ewsa/ewsa* mutants contained more chondrocytes than the 4 dpf *wt/wt* embryos (Fig. 3B). One possible explanation for the small size and high numbers of chondrocytes in the MZ *ewsa/ewsa* mutants is that the cells fail to mature into hypertrophic chondrocytes. To address this possibility, we performed *in situ* hybridization using probes for *ihha* (a marker for prehypertrophic chondrocytes) and *colX* (a marker for hypertrophic chondrocytes) in 4 dpf *wt/wt* and MZ *ewsa/ewsa* mutant [45]. As a result, there was an increase of *ihha* mRNA level in Meckel’s cartilage (100%, n = 10) in MZ *ewsa/ewsa* mutant compared to *wt/wt* (n = 9) (Fig. 3C). On the other hand, there was a reduction of *colX* mRNA in MZ *ewsa/ewsa* mutant (100%, n = 9) compared to *wt/wt* (n = 9) (Fig. 3C). We also performed immunohistochemistry using an anti-Indian hedgehog (IHH) antibody, a marker for prehypertrophic chondrocytes. As a result, 100% of 4 dpf MZ *ewsa/ewsa* mutants (n = 11) and only 12% of *wt/wt* embryos (n = 10) displayed a high number and strong signal for IHH-positive cells (Fig. 3D). To further confirm this result, Collagen type X, a marker for hypertrophic chondrocytes, was visualized by immunohistochemistry in the 5 dpf MZ *ewsa/ewsa* mutants and *wt/wt* embryos (Fig. 3D). As a result, we observed the downregulation of Collagen type X in all of the MZ *ewsa/ewsa* mutants (n = 9) compared with the *wt/wt* embryos (n = 10) (Fig. 3D). Because there is a higher numbers of prehypertrophic chondrocytes in the MZ *ewsa/ewsa* mutants, these results suggest that Ewsa promotes differentiation of prehypertrophic chondrocytes into hypertrophic chondrocytes. However, we cannot exclude the possibility of aberrant connective tissue formation leading to the misaligned chondrocytes in the MZ *ewsa/ewsa* mutant.

### Ewsa interacts with Sox9

Craniofacial cartilages are neural crest-derived tissues, and their differentiation is regulated by SOX9 at the transcriptional level [21,22,27,46]. Sox9 is a transcription factor that plays a key role in prehypertrophic chondrocyte and chondrocyte differentiation during endochondral ossification by regulating its target genes (e.g., *COL2*, *CTGF*, *NOGGIN*, and *BMP*) [26,28,31,47–49]. In a previous study, *SOX9/wt* mutant mice developed an expanded hypertrophic zone, and this aberrant phenotype was rescued by knock-in of *SOX9* [29,34]. These results suggest that SOX9 inhibits the differentiation of prehypertrophic chondrocytes into hypertrophic chondrocytes. Interestingly, similar cell morphological changes have been reported using a morpholino against Sox E3 (Sox9 homologue) in Sea lamprey, suggesting the conserved function of SOX9 among species [49]. Because MZ *ewsa/ewsa* zebrafish mutant displayed a reduced hypertrophic chondrocyte zone, and because EWS has been shown to be a transcriptional modulator, we hypothesized that EWS directly interacts with SOX9 and modulates its target genes during skeletogenesis.

**Figure 4:**
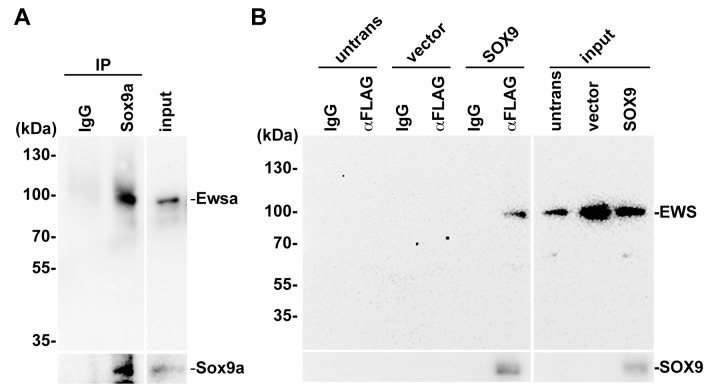
Sox9 interacts with Ewsa in zebrafish, and human SOX9 interacts with human EWS in HeLa cells. **A.** Immunoprecipitation of the lysates obtained from 27 hpf zebrafish embryos using an IgG control and anti-SOX9 antibodies. Top panel: Blot with anti-Ewsa antibody (Input: 1/660). Bottom panel: Blot with anti-Sox9 antibody (Input: 1/70). **B.** Immunoprecipitation of lysates obtained from untransfected (untrans), empty vector-transfected (vector) and *FLAG-SOX9-*transfected HeLa cells using an IgG control and anti-FLAG M2 antibody. Bottom Panel: Blot with anti-SOX9 antibody. Top Panel: Blot with anti-EWS antibody (Input: 1/200).

To determine whether Ewsa interacts with Sox9, we performed a co-immunoprecipitation (co-IP) experiment using 27 hpf zebrafish *wt/wt* embryos. First, we tested whether commercially purchased anti-SOX9 (antibody raised against human SOX9) antibody recognizes the zebrafish Sox9 protein. FLAG-tagged *sox9a* and *sox9b* DNA constructs were transfected into HeLa cells, and the cell lysates were then subjected to western blot using an anti-FLAG antibody and anti-Sox9 antibody (S3 Fig.). As a result, the anti-Sox9 antibody only recognizes ~70kDa protein that matches to the size of Sox9a (S3 Fig. Left). Consistently, in the BLAST search using the antigen site as a query, zebrafish Sox9a had the highest similarity. These results suggest that the anti-Sox9 antibody specifically recognizes zebrafish Sox9a protein. The lysates from the 27hpf embryos were prepared and subjected to immunoprecipitation using an anti-SOX9 (antibody raised against human SOX9) antibody and an anti-mouse IgG antibody as a negative control. First, the immunoprecipitation of Sox9a proteins was confirmed by western blot using an anti-SOX9 antibody (Fig. 4A bottom panel). The membrane was then subjected to western blotting using an anti-Ewsa antibody to reveal the interaction between Sox9a and Ewsa. As a result, the co-immunoprecipitation experiment revealed that Ewsa co-precipitated with Sox9a, which indicates a biochemical interaction between Ewsa and Sox9 (Fig. 4A top panel). To examine whether Sox9a and Ewsa were co-expressed in the same cells, immunohistochemistry was performed on 27 hpf *wt/wt* zebrafish embryos using anti-SOX9 and anti-Ewsa antibodies. Consistent with the results from *in situ* hybridization, Ewsa protein was expressed ubiquitously, and its localization overlapped with Sox9a (S4 Fig.). These data suggest that Ewsa may directly regulate Sox9a activity.

To further determine whether the interaction between EWS and SOX9 is conserved between zebrafish and human cells, we performed a co-IP experiment for human EWS and SOX9 using HeLa cells. A FLAG-tagged *SOX9* DNA construct was transfected into HeLa cells, and the cell lysates were extracted. These lysates were then subjected to immunoprecipitation using an anti-FLAG antibody and an anti-mouse IgG antibody as a negative control. Using an anti-SOX9 antibody, the immunoprecipitation of FLAG-tagged SOX9 proteins was confirmed by western blot analysis (Fig. 4B; bottom panel). To reveal the interaction between SOX9 and EWS, the membrane was subjected to western blotting using an anti-EWS antibody. EWS was precipitated with SOX9 indicating a biochemical interaction between EWS and SOX9 (Fig. 4B; top panel). These data indicates that the interaction between EWS and Sox9 is conserved between zebrafish and humans.

### Ewsa transcriptionally regulates Sox9 target genes

**Figure 5:**
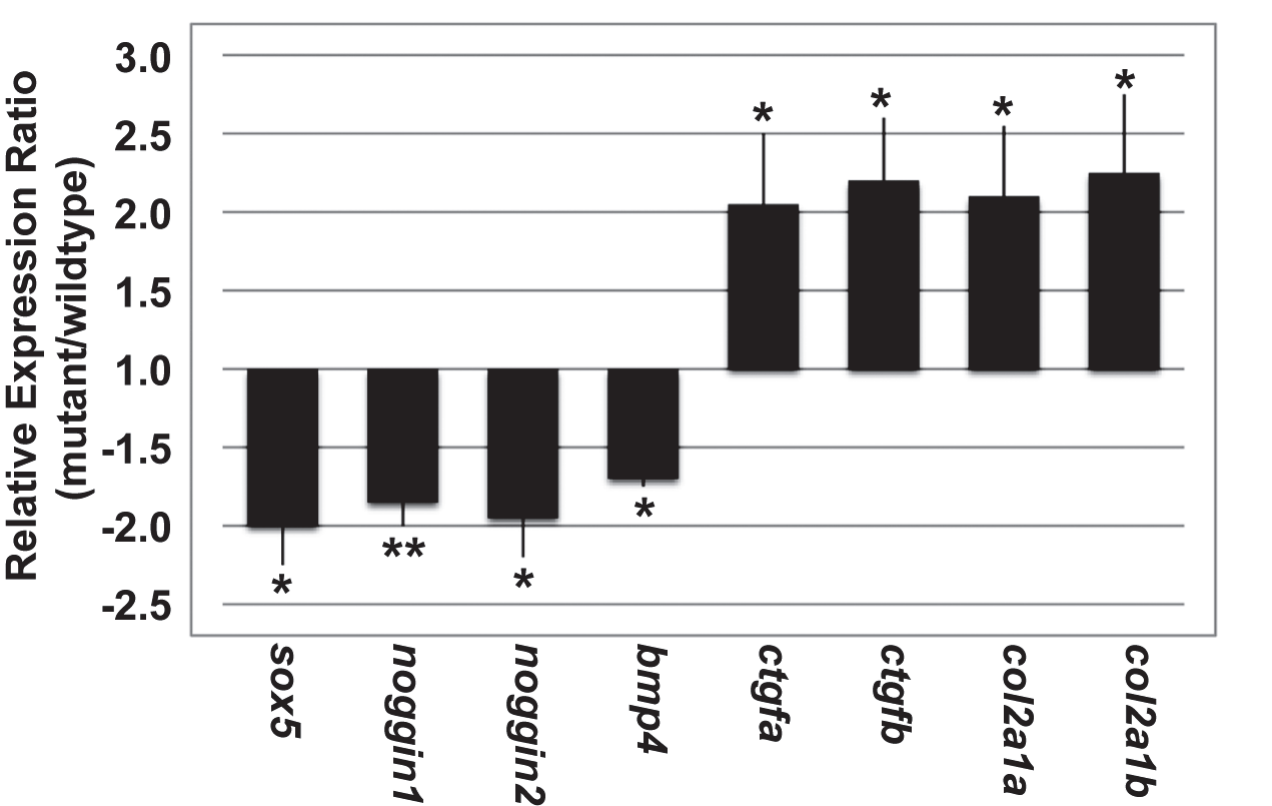
MZ *ewsa/ewsa* mutants display altered expression levels of Sox9 target genes. The ratios of the Sox9 target mRNA expression levels between the *wt/wt* and MZ *ewsa/ewsa* mutants (27 hpf) are shown in a bar plot (* p<0.05 and ** p<0.01).

To address whether Ewsa regulates the transcription of Sox9 target genes, mRNA was purified from 27 hpf *wt/wt* and MZ *ewsa/ewsa* embryos. cDNA was synthesized from the mRNA, and the expression of Sox9 target genes was quantified by qPCR. We chose Sox9 target genes from previous study that showed Sox9 binding peak on ChIP-on-ChIP experiment in rat chondrosarcoma (RCS) cell line (the gene list is shown in S1 Table) [50]. Among these genes, we found that *sox5*, *noggin1*, *noggin2*, and *bmp4* were significantly downregulated and that *ctgfa*, *ctgfb*, *col2a1a*, *and col2a1b* were significantly upregulated in the MZ *ewsa/ewsa* mutants relative to *wt/wt* (Fig. 5 and S2 Table). The relative expression and p-values calculated using Welch’s t-test are listed in S2 Table. With a significance threshold of p<0.05, 1/20 (or 1.5/30) of the genes tested at random might show significant changes in expression. However, by selecting genes that were suspected to be under Sox9 transcriptional regulation and finding that eight of the thirty genes changed significantly, we detected a relationship far beyond what could be expected by chance. It is noteworthy that there were no significant difference in the expression levels of *sox9a* and *sox9b* between 27hpf *wt/wt* and MZ *ewsa/ewsa* zebrafish embryos. These data suggest that Ewsa regulates the mRNA expression of Sox9 target genes. The results suggest that the misexpression of Sox9-target genes in the MZ *ewsa/ewsa* mutants already starts at 27hpf, and this may be the direct cause for inhibition of differentiation prehypertrophic chondrocyte observed at 4dpf.

**Figure 6:**
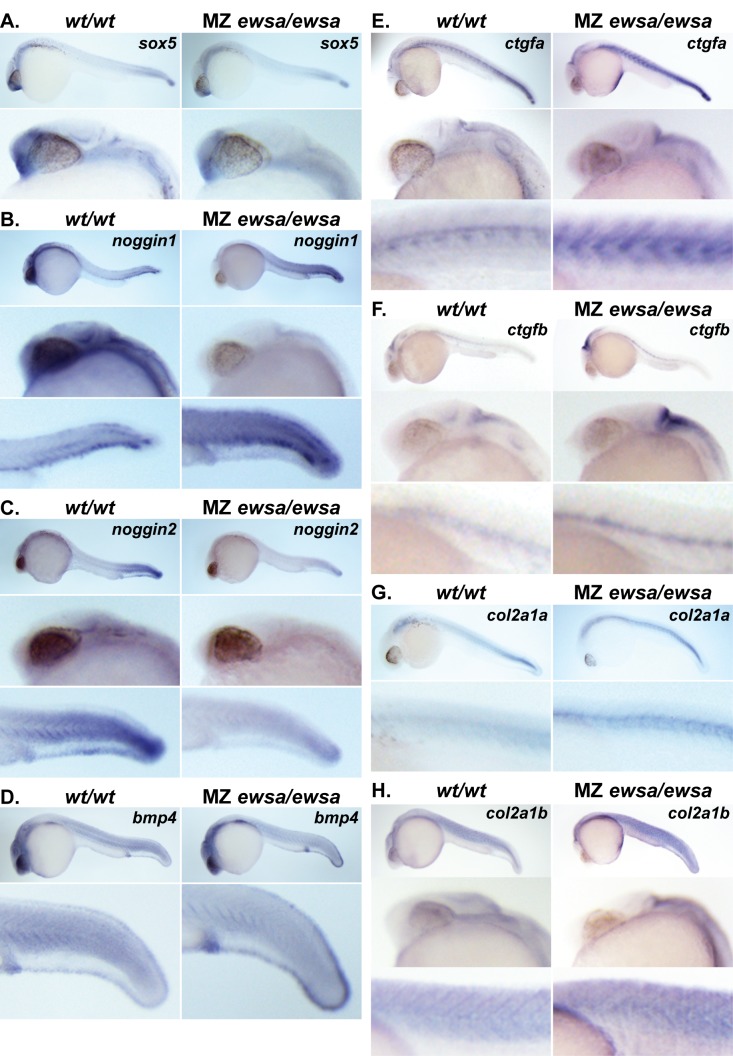
MZ *ewsa/ewsa* mutants display altered expression domains of Sox9 target genes. 27hpf of (Left) *wt/wt* and (Right) MZ *ewsa/ewsa* visualized by *in situ* hybridization using antisense RNA probe for **A.** *sox5*, **B.** *noggin1*, **C.** *noggin2*, **D.** *bmp4*, **E.** *ctgfa*, **F.** *ctgfb*, **G.** *col2a1a* and **H.** *col2a1b*. Top panel: low magnification images, middle and bottom panel: high magnification images.

We further performed *in situ* hybridizations using probes against eight genes that showed the significant expression change in *wt/wt* and MZ *ewsa/ewsa* mutant embryos (Fig. 6 and S3 Table). Consistent with Fig. 5, expression of *sox5*, *noggin1*, and *noggin2*, were downregulated and *ctgfa*, *ctgfb*, *col2a1a*, *and col2a1b* were upregulated in the MZ *ewsa/ewsa* mutants compared to *wt/wt*. Only the expression of *bmp4* was inconsistent with the result of qPCR, displaying a slight upregulation in the MZ *ewsa/ewsa* mutants. The reason for discrepancy between these two experiments is unknown. One explanation may be that qPCR experiment was designed to amplify the 5’ region of *bmp4* gene (100bp), whereas full length probe against *bmp4* gene was used for *in situ* hybridization. Because EWS can modulate splicing, 5’ region of *bmp4* gene may have been eliminated in the mRNA due to mis-splicing in the mutant, and it may be the cause for the reduction signal of qPCR in the mutant.

**Figure 7:**
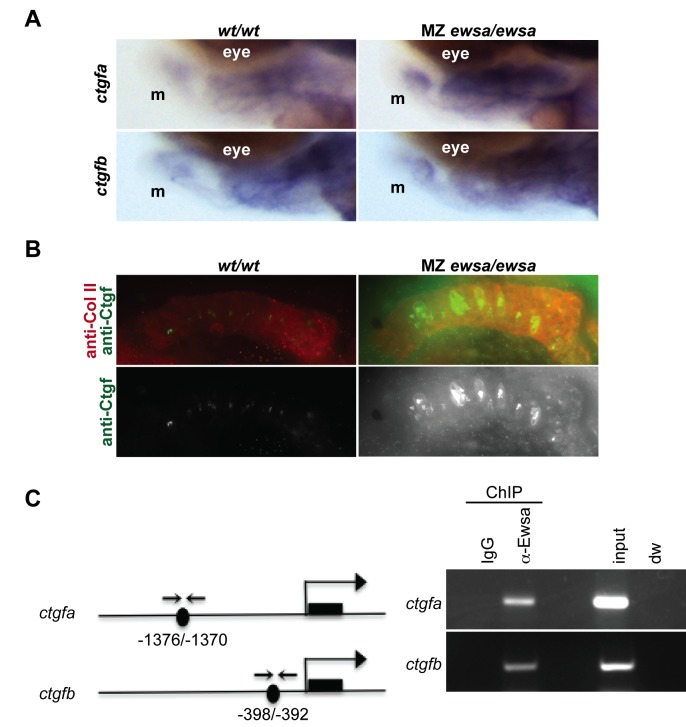
Ewsa binds to *ctgfa* and *ctgfb* target genes. **A.** Lateral views (anterior to the left, dorsal to the top) of 4dpf of (Left) *wt/wt* and (Right) MZ *ewsa/ewsa* visualized by *in situ* hybridization using probe for *ctgfa* (tom panel) and *ctgfb* (bottom panel). m: Meckel's cartilage. **B.** Ventral views (anterior to the left) of 4dpf Meckel's cartilage of (Left) *wt/wt* and (Right) MZ *ewsa/ewsa* visualized by immunohistochemistry using anti-Ctgf antibody (green), and anti-Collagen type II antibody (Red). **C.** (left) Schematics of *ctgfa* and *ctgfb* genes. Black circle: Sox9 binding site, black square: exon, arrows: PCR primer for ChIP assay. (right) ChIP assays were performed using 27 hpf zebrafish embryos. IgG: negative control of ChIP, anti-Ewsa: ChIP sample using EWSa antibody, input: 4.5% of DNA from total lysates was subjected to PCR, dw: negative control for PCR reaction.

To specify which Sox9-target genes obtained from Fig. 5 and 6 are responsible for the inhibition of differentiation in the craniofacial prehypertrophic chondrocytes, Ctgf was chosen as a candidate that is responsible for the phenotypic change in MZ *ewsa/ewsa* mutant because it regulates differentiation of prehypertrophic chondrocytes into hypertrophic chondrocytes [35]. Consistent with Fig. 5 and 6, *in situ* hybridizations using probes against *ctgfa* displayed increased expression in MZ *ewsa/ewsa* (100%, n = 11) mutant embryos compared to *wt/wt* at 3dpf (n = 11) (Fig. 7A). On the other hand, *ctgfb* mRNA level was similar between both genotypes (n = 10) (Fig. 7A). It is possible that Ewsa modulates the expression level of *ctgfb* at 27hpf as shown in Fig. 5 and 6, but not at 3dpf. To examine whether Ctgf protein level is also increased in the mutant, we further performed immunohistochemistry using antibody against Ctgf protein. For this experiment, we used a commercially available anti-CTGF antibody that was generated against mouse CTGF. For the BLAST search using the antigen site of mouse CTGF as a query, zebrafish Ctgfa had the highest identity (81%), whereas Ctgfb had lower identity (61%). For this reason, the signal detected by immunohistochemistry using the anti-CTGF antibody is most likely zebrafish Ctgfa. Ctgf displayed a high level of signal intensity in the craniofacial chondrocytes in all of the 4 dpf MZ *ewsa/ewsa* mutants (n = 9), whereas, only 22% of *wt/wt* (n = 9) displayed high level of Ctgf signal intensity (Fig. 7B). Expression level of Ctgf is known to be downregulated in the hypertrophic chondrocytes. The high level of Ctgf may be a major cause for the high number of prehypertrophic chondrocytes and the inhibition of their differentiation. These data suggest that the Ewsa regulates chondrogenesis through inhibiting the expression of Ctgf during the transition from prehypertrophic chondrocytes to hypertrophic chondrocytes.

Because Ewsa inhibits expression of *ctgfa* and *ctgfb* mRNA at 27hpf embryo (Fig. 5A), we further aimed to elucidate whether Ewsa directly interacts with promoter sequences of *ctgf* genes. The PCR primers were designed at the Sox9 consensus sequence of *ctgfa* and *ctgfb* promoters, and ChIP assay was performed in 27hpf *wt/wt* zebrafish embryo (Fig. 7C). As a result, Ewsa directly interacted with the *ctgfa* and *ctgfb* promoters that contain Sox9 consensus sequences (Fig. 7C). Together, with the direct interaction between Ewsa and Sox9, these data suggest that Ewsa may directly regulate Sox9 at the Sox9 target loci. This study is the first demonstration of Ewsa-dependent Sox9 regulation during chondrogenesis.

## Discussion

To elucidate the role of Ewsa in skeletal development, we investigated the phenotype and its mechanistic cause in MZ *ewsa/ewsa* zebrafish mutants. The Meckel’s cartilage in the MZ *ewsa/ewsa* mutants displayed a greater number of prehypertrophic chondrocytes and failed to differentiate into hypertrophic chondrocytes compared with the *wt/wt* embryos. These results indicate that Ewsa regulates chondrogenesis/skeletogenesis by promoting the differentiation of prehypertrophic chondrocytes into craniofacial hypertrophic chondrocytes. We discovered that Sox9 and Ewsa biochemically interact with each other, and that Ewsa interacts with Sox9 target loci and transcriptionally modulates the mRNA levels. Taken together, we propose that Ewsa regulates chondrogenesis/skeletogenesis by modulating the transcriptional activity of SOX9.

Our findings provide a molecular basis of EWS function during chondrogenesis/ skeletogenesis and may help to explain why Ewing sarcoma specifically develops in skeletal tissue. Ewing sarcoma cells display an undifferentiated cell morphology called small round blue cells, suggesting that their differentiation is impaired. Because the MZ *ewsa/ewsa* mutants failed to mature into hypertrophic chondrocytes and accumulate prehypertrophic chondrocytes, it is conceivable that the impairment of EWS may contribute to Ewing sarcoma development. One explanation for the pathogenesis is that the inhibition of prehypertrophic chondrocyte differentiation increases the incidence of DNA mutation. Mature hypertrophic chondrocytes do not undergo the cell cycle because these cells are terminally differentiated. In contrast, prehypertrophic chondrocytes are capable of proliferating. Therefore, the prehypertrophic chondrocytes in MZ *ewsa/ewsa* mutants may have entered the cell cycle as we observed in a higher number of cells (Fig. 3A and B), which may increase the incidence of DNA mutations due to EWS knockdown-dependent cell cycle defects. Indeed, multiple studies have reported that the inhibition of EWS may lead to induction of DNA mutations. We previously reported that EWS interacts with the key mitotic regulator, Aurora B kinase. The EWS-Aurora B interaction is required for midzone formation that is essential for cytokinesis, a stage of cell division [17]. Compromising midzone formation has been shown to lead to higher incidences of aneuploidy [51]. Other reports have shown that the EWS knockdown leads to the alternative splicing of DNA repair genes [52]. Supporting this hypothesis, Ewing sarcoma cells only retained the single EWS allele due to the formation of EWS/FLI1, which may result in reduced EWS protein levels. In addition, EWS/FLI1 interacts with EWS and inhibits the function of EWS during mitosis, suggesting that EWS/FLI1 may also play a dominant role in EWS-dependent Sox9 regulation [17,19]. Importantly, this study provides a significant platform to study the function of EWS/FLI1 during chondrogenesis.

The qPCR analysis demonstrated that Ewsa regulates Sox9 target genes by either inhibiting (*ctgfa*, *ctgfb*, *col2a1a*, and *col2a1b*) or activating (*sox5*, *nog1*, *nog2*, and *bmp4*) their transcription in the *wt/wt* zebrafish embryos. This observation was supported by the direct interaction between Ewsa and Sox9a. In a future study, identifying the mechanism for Ewsa-dependent activation or inhibition of Sox9 transcriptional activity is essential. A possible mechanism may be that the transcriptional activity could be altered in a co-factor dependent manner. A potential candidate for the gene activation may be p300 because it has been individually reported to associate with and activate transcription with EWS and SOX9 [53]. EWS, SOX9, and p300 may form a complex, or EWS/p300 and SOX9/p300 heterodimers may compete for target loci [54,55]. In addition, determining the inhibitory co-factor for the transcriptional activity of SOX9 target genes is yet to be determined, but this co-factor may be determined by identifying the component in the complex obtained from ChIP samples using an Ewsa antibody. It is also necessary to perform these experiments using chondrocytes.

This study is the first demonstration of EWS-dependent Sox9 regulation during skeletogenesis. We identified that Ewsa regulates *ctgfa* and *ctgfb* genes through direct interaction with the promoter region of these genes. This regulation may be essential for the differentiation of prehypertrophic chondrocytes to hypertrophic chondrocytes in Meckel’s cartilage. This study may provide a platform for dissecting the EWS/FLI1 pathway in skeletal cells and to determine how skeletal differentiation is impaired in Ewing sarcoma cells in future studies, and it may provide insight into the formation of Ewing sarcoma.

## Supporting Information

S1 Fig The MZ *ewsa/ewsa* mutant displays normal patterning. (TIF)

S2 Fig The MZ *ewsa/ewsa* mutant displays normal patterning of craniofacial chondrocytes. (TIF)

S3 Fig The Sox9 antibody recognizes zebrafish Sox9a. (TIF)

S4 Fig Sox9 colocalizes with Ewsa at 27 hpf. (TIF)

S1 Table List of Sox9 target genes that were tested in *wt/wt* and MZ *ewsa/ewsa* zebrafish mutant. *: Genes with a significant difference in relative fold expression of mutant/wildtype.
(DOCX)

S2 Table Relative fold gene expression in MZ *ewsa/ewsa* mutants compared to the wild type. Relative fold gene expression: mutant/wildtype.
(DOCX)

S3 Table MZ *ewsa/ewsa* mutants display altered expression of Sox9 target genes. Numbers of embryo with normal expression / total numbers of embryo.
(DOCX)
