# Correction: Ewing Sarcoma Ewsa Protein Regulates Chondrogenesis of Meckel's Cartilage through Modulation of Sox9 in Zebrafish

**DOI:** 10.1371/journal.pone.0123487

**Published:** 2015-03-30

**Authors:** The PLOS ONE Staff

There is an error in the Funding section. The correct funding information is as follows: This manuscript was supported by the Massman Family Ewing Sarcoma Research Fund, the Sarcoma Foundation of America, P20RR016475 / P20GM103418 and P20GM103638. The funders had no role in study design, data collection and analysis, decision to publish, or preparation of the manuscript.
