# Supplementary figures and images for: Ewing Sarcoma Ewsa Protein Regulates Chondrogenesis of Meckel’s Cartilage through Modulation of Sox9 in Zebrafish
Source: PLoS One. 2015 Jan 24;10(1):e0116627. doi: 10.1371/journal.pone.0116627 (PMC4305327; doi:10.1371/journal.pone.0116627)

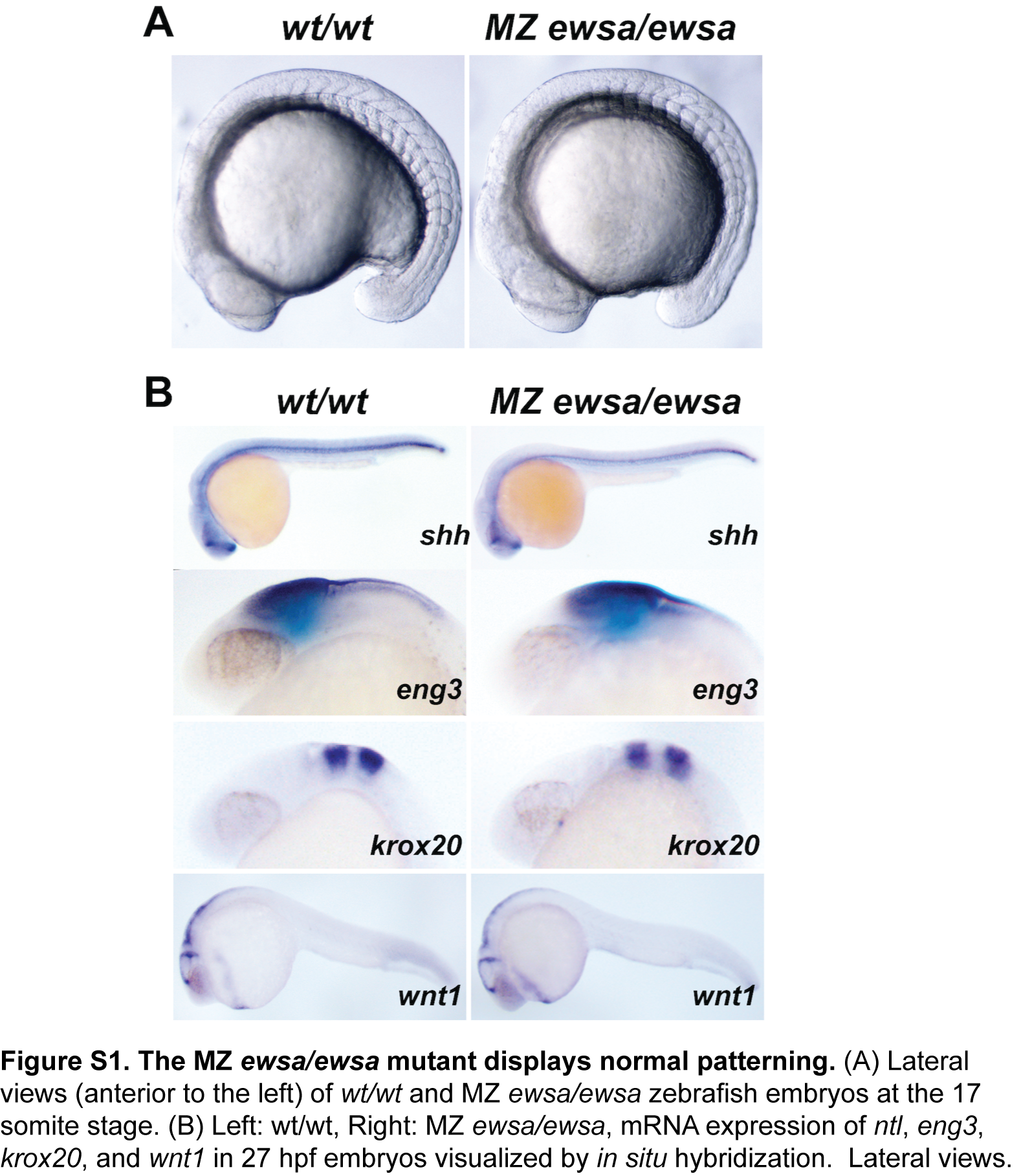

Supplement: S1 Fig — (TIF) [file pone.0116627.s001.tif]

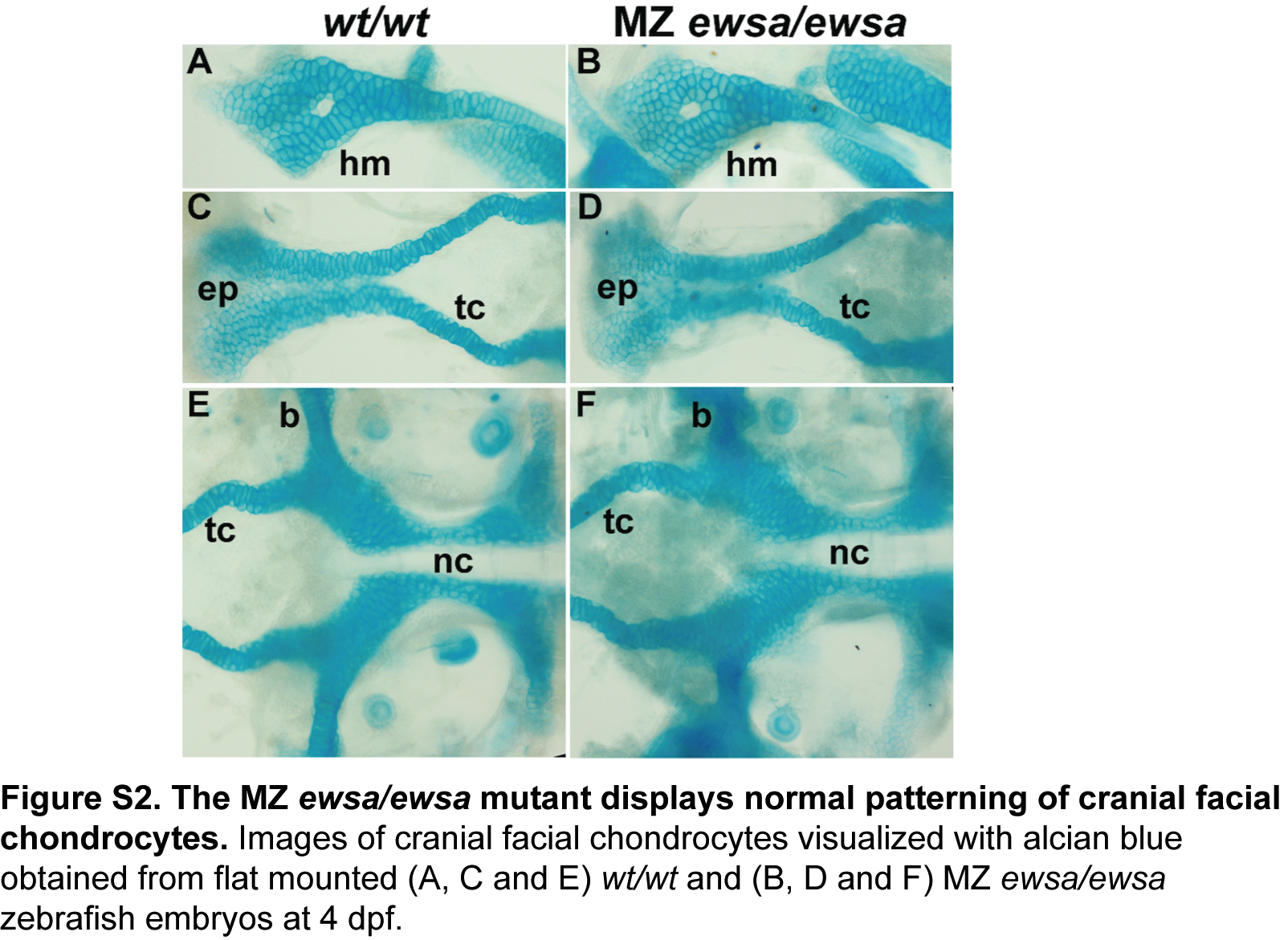

Supplement: S2 Fig — (TIF) [file pone.0116627.s002.tif]

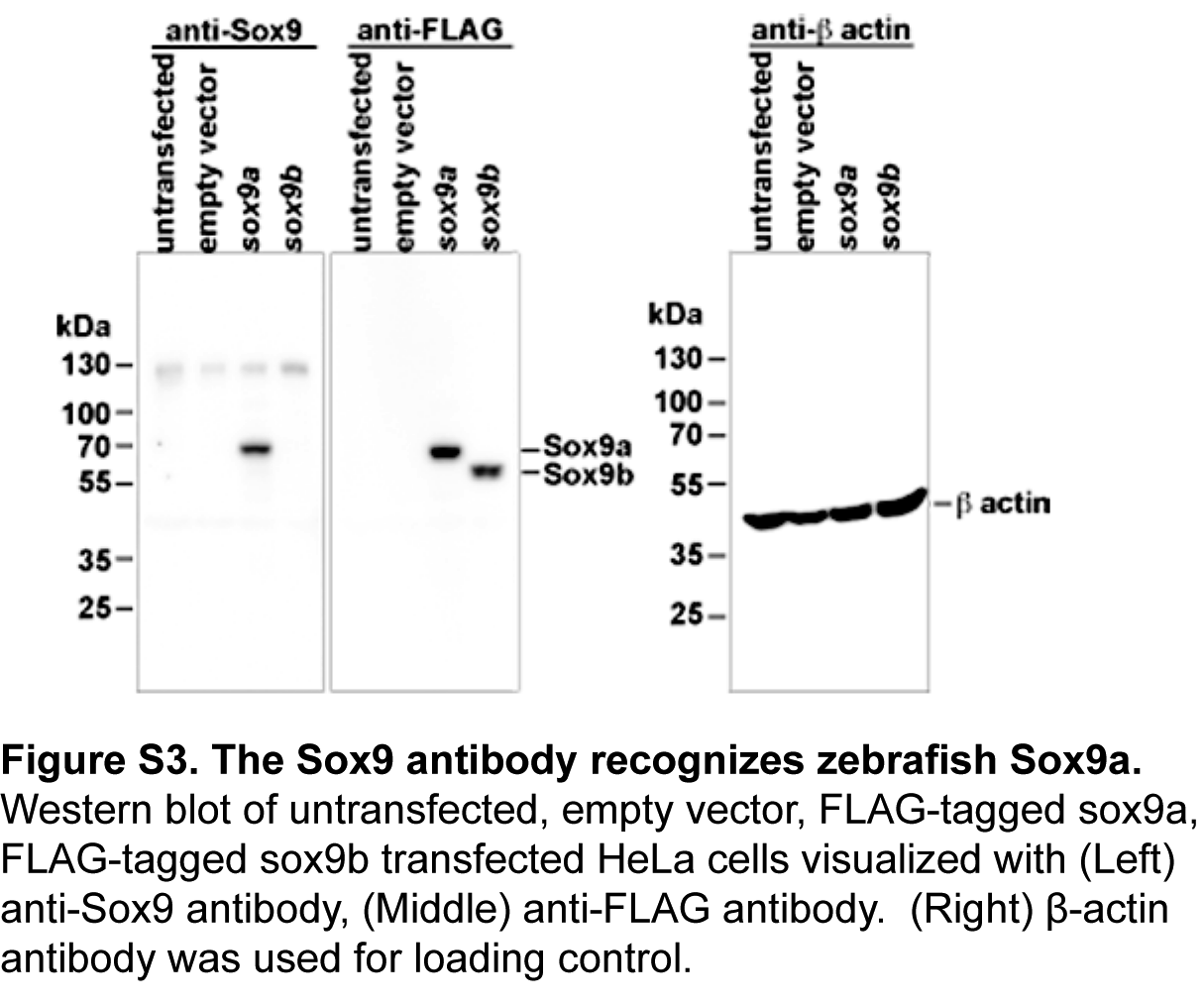

Supplement: S3 Fig — (TIF) [file pone.0116627.s003.tif]

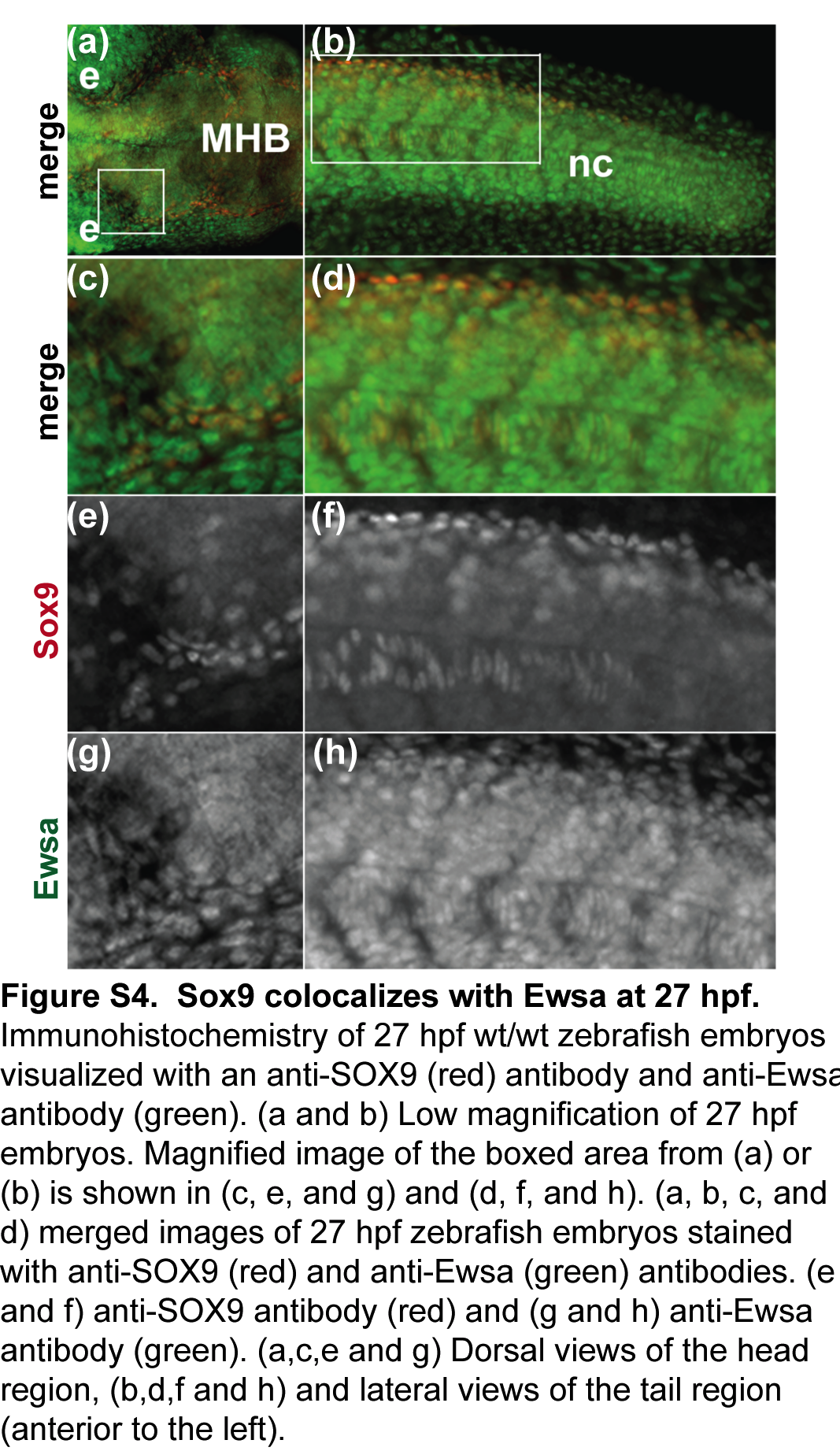

Supplement: S4 Fig — (TIF) [file pone.0116627.s004.tif]
