# Supplementary material for: Ewing Sarcoma Ewsa Protein Regulates Chondrogenesis of Meckel’s Cartilage through Modulation of Sox9 in Zebrafish
Source: PLoS One. 2015 Jan 24;10(1):e0116627. doi: 10.1371/journal.pone.0116627 (PMC4305327; doi:10.1371/journal.pone.0116627)
Supplement: S1 Table — *: Genes with a significant difference in relative fold expression of mutant/wildtype. (DOCX) [file pone.0116627.s005.docx]

**Table S1: List of Sox9 target genes that were tested in *wt/wt* and MZ *ewsa/ewsa* zebrafish mutant.** *: Genes with a significant difference in relative fold expression of mutant/wildtype.

| **Gene** |
| --- |
| ** bmp4 (bone morphogenetic protein 4)* |
| *cata 1* |
| *cata 2* |
| ** col1a1a (Collagen type I alpha 1)* |
| ** col2a1a (Collagen type II alpha 1)* |
| ** col2a1b (Collagen type II beta 1)* |
| *col9a2 (Collagen type VIIII alpha 2)* |
| *col11a1a (Collagen type XI alpha 1)* |
| *col11a1b (Collagen type XI beta 1)* |
| *col11a2 (Collagen type XI alpha 1)* |
| ** ctgfa (connective tissue growth factor a)* |
| ** ctgfb (connective tissue growth factor b)* |
| *ctsb (cathepsin)* |
| *epyc (epiphycan)* |
| *erk1 (extracellular signal-regulated protein kinase 1)* |
| *erk2 (extracellular signal-regulated protein kinase 1)* |
| *fmoda (fibromodulin a)* |
| *grb10a (growth factor receptor-bound protein 10)* |
| *igf2r (insulin-like growth factor 2 receptor)* |
| *lef1 (lymphoid enhancer binding factor 1)* |
| *matn4 (matrilin 4)* |
| ** noggin1* |
| ** noggin 2* |
| *ptch1 (patched homolog 1)* |
| *ptch2 (patched homolog 2)* |
| *prelp (proline/arginine-rich end leucine-rich repeat protein)* |
| *prkacaa (protein kinase, cAMP-dependent, catalytic, alpha)* |
| *prkacab (protein kinase, cAMP-dependent, catalytic, beta)* |
| *runx2 (runt related transcription factor 2)* |
| *sdc3 (syndecan 3)* |
| ** sox5 (SRY (sex determining region Y)-box 5)* |
| *sox9a (SRY (sex determining region Y)-box 9 a)* |
| *sox9b (SRY (sex determining region Y)-box 9 b)* |
| *stat 1a (signal transducer and activator of transcription 1 a)* |
| *tgfb3 (transforming growth factor, beta 3)* |
| *vegfa (vascular endothelial growth factor a)* |
| *vegfb (vascular endothelial growth factor b)* |
