# Supplementary material for: Ewing Sarcoma Ewsa Protein Regulates Chondrogenesis of Meckel’s Cartilage through Modulation of Sox9 in Zebrafish
Source: PLoS One. 2015 Jan 24;10(1):e0116627. doi: 10.1371/journal.pone.0116627 (PMC4305327; doi:10.1371/journal.pone.0116627)
Supplement: S2 Table — Relative fold gene expression: mutant/wildtype. (DOCX) [file pone.0116627.s006.docx]

**Table S2: Relative fold gene expression in MZ *ewsa/ewsa* mutants compared to the wild type.** Relative fold gene expression: mutant/wildtype.

| **mRNA** | **Relative Fold Expression** | **P-value** |
| --- | --- | --- |
| ***sox5*** | - 2.0 | 0.03 |
| ***noggin 1*** | - 1.7 | 0.006 |
| ***noggin 2*** | - 1.9 | 0.03 |
| **bmp 4** | - 1.4 | 0.01 |
| ***ctgfa*** | + 2.1 | 0.03 |
| ***ctgfb*** | + 2.4 | 0.02 |
| ***col2a1a*** | + 2.2 | 0.04 |
| ***col2a1b*** | + 2.5 | 0.03 |
