# Supplementary material for: Ewing Sarcoma Ewsa Protein Regulates Chondrogenesis of Meckel’s Cartilage through Modulation of Sox9 in Zebrafish
Source: PLoS One. 2015 Jan 24;10(1):e0116627. doi: 10.1371/journal.pone.0116627 (PMC4305327; doi:10.1371/journal.pone.0116627)
Supplement: S3 Table — Numbers of embryo with normal expression / total numbers of embryo. (DOCX) [file pone.0116627.s007.docx]

**Table S3: MZ *ewsa/ewsa* mutants display altered expression of Sox9 target genes.** Numbers of embryo with normal expression / total numbers of embryo.

| **probe** | ***wt/wt*** (normal/total) | **MZ *ewsa/ewsa*** (normal/total) |
| --- | --- | --- |
| ***sox5*** | 10/10 | 3/13 |
| ***noggin 1*** | 8/9 | 1/9 |
| ***noggin 2*** | 8/9 | 1/9 |
| **bmp 4** | 8/8 | 1/12 |
| ***ctgfa*** | 12/12 | 0/8 |
| ***ctgfb*** | 17/17 (notochord)  15/17 (hindbrain) | 3/9 (notochord)  0/9 (hindbrain) |
| ***col2a1a*** | 20/20 | 1/26 |
| ***col2a1b*** | 14/14 | 0/16 |
